# Copper-Guanosine Nanorods (Cu-Guo NRs) as a Laccase Mimicking Nanozyme for Colorimetric Detection of Rutin

**DOI:** 10.3390/bios13030374

**Published:** 2023-03-12

**Authors:** Kowsar Davoodi-Rad, Ardeshir Shokrollahi, Faezeh Shahdost-Fard, Kamal Azadkish

**Affiliations:** 1 Chemistry Department, Yasouj University, Yasouj 75914-353, Irank.azadkish@gmail.com (K.A.); 2 Department of Chemistry, Farhangian University, Tehran 19396-14464, Iran

**Keywords:** nanozyme, rutin, copper-guanosine nanorods, laccase mimicking, colorimetric, smartphone-based sensing

## Abstract

Inspired by laccase activity, herein, Cu-guanosine nanorods (Cu-Guo NRs) have been synthesized for the first time through a simple procedure. The activity of the Cu-Guo NR as the laccase mimicking nanozyme has been examined in the colorimetric sensing of rutin (Rtn) by a novel and simple spectrophotometric method. The distinct changes in the absorbance signal intensity of Rtn and a distinguished red shift under the optimum condition based on pH and ionic strength values confirmed the formation of the oxidized form of Rtn (o-quinone) via laccase-like nanozyme activity of Cu-Guo NRs. A vivid and concentration-dependent color variation from green to dark yellow led to the visual detection of Rtn in a broad concentration range from 770 nM to 54.46 µM with a limit of detection (LOD) of 114 nM. The proposed methodology was successfully applied for the fast tracing of Rtn in the presence of certain common interfering species and various complex samples such as propolis dry extract, human biofluids, and dietary supplement tablets, with satisfactory precision. The sensitivity and selectivity of the developed sensor, which are bonuses in addition to rapid, on-site, cost-effective, and naked-eye determination of Rtn, hold great promise to provide technical support for routine analysis in the real world.

## 1. Introduction

Rutin (Rtn), a plant-derived polyphenolic bioflavonoid, is a therapeutically active phytochemical [1]. Hydroxyl groups in the Rtn structure are a benchmark for its potent biological properties, especially antioxidant activities [2,3]. Rtn is an adjunct therapy for hypertensive encephalopathy, postpartum hemorrhage, retinal hemorrhage, and cerebral accidents [4,5,6,7,8,9] and has a potential protective role in some neurodegenerative diseases (NDs) induced by aging [10,11]. Furthermore, it has electroactive property and offers various applications in electrochemical studies [12,13]. So, monitoring and tracing this natural health protector in different samples is medically crucial. Various analytical methods have been introduced so far for Rtn determination, including high-performance liquid chromatography (HPLC) [14], capillary electrophoresis [15], chemiluminescence [16], flow injection analysis [17], and electrochemistry [18]. Despite the accuracy of some of them, these methods are mainly limited due to being costly, time-consuming, or requiring tedious sampling equipment, sophisticated instrumentation, and trained technicians [1,19,20,21,22,23,24,25,26,27,28]. Therefore, developing a rapid, easy-to-use, and cost-effective sensor for on-site monitoring of Rtn is highly desirable. 

Due to its high sensitivity and wide linear range, sensory evaluation has recently become an effective tool in biomarker detection and clinical diagnosis. For example, creatinine, alanine aminotransferase, acetylcholine, cholesterol, and p-cresol have been successfully detected using localized surface plasmon resonance (LSPR) based on spectrophotometric methods [29,30,31,32,33]. Although spectrophotometric-based procedures are easy to use, they are rarely reported for Rtn measurement [20]. Optical sensors based on catalyzing phenolic compounds are inexpensive tools for the selective detection of the analyte [34,35]. The applicability of this type of colorimetric sensor is boosted when their response could be directly read out with the naked eye. These fascinating and inexpensive simplified tools have been applied for fast visual detection of various analytes [36,37]. 

Laccases are a family of multi-copper (Cu) enzymes that naturally oxidize various compounds of polyphenols, aryl diamines, aromatic amines, and polyamines [38,39]. Although laccases with effectual catalytic potential are considered green enzymes, some limitations such as low stability and yield, difficult storage, and high cost of their separation and purification [40] significantly hinder their application. Utilizing nanomaterials that mimic natural enzymes, called nanozymes, as beneficial catalysts may overcome these issues. Nanozymes have served as direct alternatives to traditional enzymes by engineering the active centers of natural enzymes. Some nanostructures with laccase-like activity have been introduced for various applications, namely, metal oxide nanoparticles (MeONPs) [34], Cu-carbon dots (CDs) [41], Cu/nucleotide metal-organic frameworks (MOFs) [40], Cu-tannic acid nanohybrids [42], and DNA-Cu hybrid nanoflowers (NFs) [43].

In this study, inspired by the active site of laccase, a nanozyme based on Cu-guanosine nanorods (NRs), defined as Cu-Guo NRs, has been greenly synthesized for the first time and applied for the colorimetric sensing of Rtn. By tuning the reaction condition based on pH and ionic strength, a vivid ranging colorimetric response from various tonalities of light green to dark yellow was obtained for different concentrations of Rtn. The proposed methodology has been successfully applied for a relatively fast Rtn assay in various complex samples such as dry propolis extract, human biofluids and dietary supplement tablets with satisfactory precision and accuracy. To the best of our knowledge, there are no reports in the literature for the simple and visual measurement of Rtn based on the proposed nanozyme strategy. 

## 2. Experimental

### 2.1. Reagents and Apparatus

Rtn (95% purity), quercetin (Qur) (98% purity), gallic acid (GA) (98% purity), ascorbic acid (AA), phytic acid, guanosine (Guo), 2,4-dichlorophenol (2,4-DP), 4-aminoantipyrine (4-AP), copper (II) sulfate pentahydrate (CuSO_4_·5H_2_O), terephthalic acid, 4-(2-hydroxyethyl)-1-piperazineethanesulfonic acid (HEPES), and other reagents of analytical grade were purchased from Sigma Aldrich and Merck Companies. Milli-Q grade deionized (DI) water with a resistivity of 18.2 MΩ was applied in all tests. 

Absorbance spectra were recorded using a Perkin-Elmer Lambda 25 UV/Vis spectrometer (Waltham, MA, USA) equipped with a 1 cm glass cell. An RHB model centrifuge (Osterode, Germany) was used for mixture separation. A smartphone camera (Xiaomi Redmi note 8 pros with a display resolution of 1080 × 2340 pixels and pixel density of 395 pixels per inch) was used for manually taking all photographic images without any filter. Transmission electron microscopic (TEM) images of the synthesized Cu-Guo NRs were recorded with a Zeiss EM900 microscope (Oberkochen, Germany) at an accelerating voltage of 200 kV. The morphology, chemical composition analysis, and elemental mapping of Cu-Guo NRs were investigated with a scanning electron microscopy (SEM) instrument equipped with an electron dispersive X-ray analyzer (EDX) on an SEM FEI Quanta 200 instrument (Eindhoven, The Netherlands). Fourier transform infrared (FTIR) spectra were recorded by a Jasco-680 FTIR spectrometer. 

### 2.2. Preparation of Cu-Guo NRs Nanozymes

**Scheme 1:**
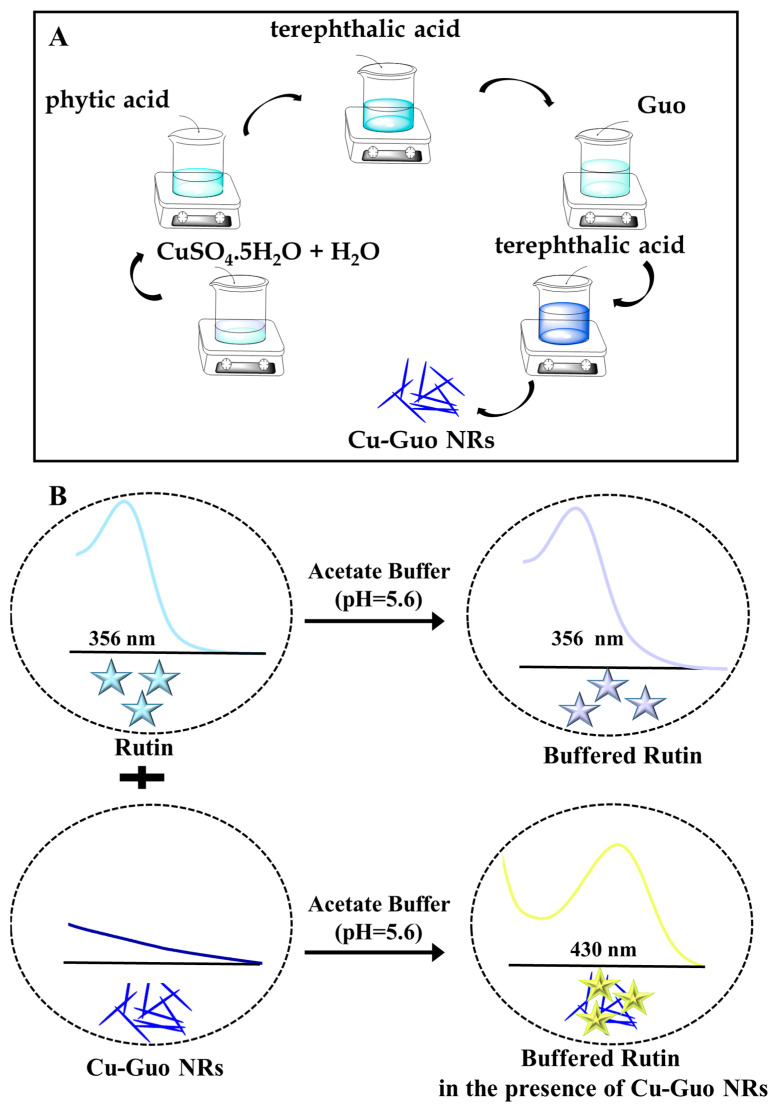
(**A**) The synthesis process of Cu-Guo NRs as the nanozyme and (**B**) the sensing strategy of the proposed sensor using Cu-Guo NRs for Rtn measurement.

The synthesis of Cu-Guo NRs was simulated according to the literature in a simple manner [34] with some modification. Briefly, CuSO_4_·5H_2_O (0.2 g) was dissolved in 10 mL of DI water, and then 5 mL of phytic acid (3 mM) was slowly added to it. Then 1 mL of terephthalic acid (0.18 mM) and 5 mL of Guo (14 mM) were gradually added to the above solution. Next, more terephthalic acid (1.8 mM, 10 mL) was added to them. After 1 h stirring and centrifuging, the final precipitate was collected and washed with DI water. The obtained product, called Cu-Guo NRs, was dried at 40 °C for 12 h and finally kept at 4 °C for further experiments (Scheme 1A). To investigate the reproducibility of the proposed sensor, different NRs were prepared in three consecutive days.

### 2.3. General Procedure for Colorimetric Detection of Rtn

The Rtn assay was readily accomplished based on the catalytic oxidation of Rtn using Cu-Guo NRs under relatively acidic conditions. The influences of several parameters on the catalytic reaction of the nanozyme, including the type of buffer, pH value, Cu-Guo NRs volume, and reaction time were studied. Under optimized conditions, a series of different concentrations of ethanolic solution of Rtn was added to a solution containing 700 μL of Cu-Guo NRs (1 mg m$L^{-1}$) and 1 mL of acetate buffer solution (300 mM). Then, the final volume of the solutions was adjusted by DI water to 3 mL. The colorimetric detection of Rtn was carried out for 6 min after adding the last drop of Rtn by recording the absorbance spectra and taking corresponding digital images through a smartphone’s camera. All measurements were conducted in triplicate at room temperature.

### 2.4. Preparation of Real Samples

Propolis, as a dry extract containing Rtn, was obtained from Zardband Pharmaceutical Company (Yasuj, Iran). First, 75 mL of ethanol was added to 100 mg of the propolis sample and stirred for 2 h. Then, it was brought to the volume of 100 mL with the same solvent, and finally it was filtered. Rtn-containing dietary supplement tablets (20 mg per tablet) were purchased from a local pharmacy. Five tablets were randomly selected and ground to obtain a fine powder. Then 100 mg of the resulting powder was dispersed in 40 mL of ethanol, and the sample was diluted by ethanol to 50 mL. Urine samples were randomly provided from two healthy 25- and 31-years old girls from Dena pathobiology laboratory at Yasuj City by observing human ethical principles. Then 10 mL of each urine sample was centrifuged at 1500 rpm for 10 min, and then 1 mL of the supernatant was diluted with 5 mL of DI water. A blood serum sample from a healthy 40-year-old man was randomly obtained from Dena pathobiology laboratory at Yasuj City by observing human ethical principles, and 1 mL of this was diluted with 10 mL of DI water. Finally, the samples were spiked with different concentrations of Rtn and tested by the proposed sensor.

## 3. Results

### 3.1. Morphology and Characterization of Cu-Guo NRs

**Figure 1:**
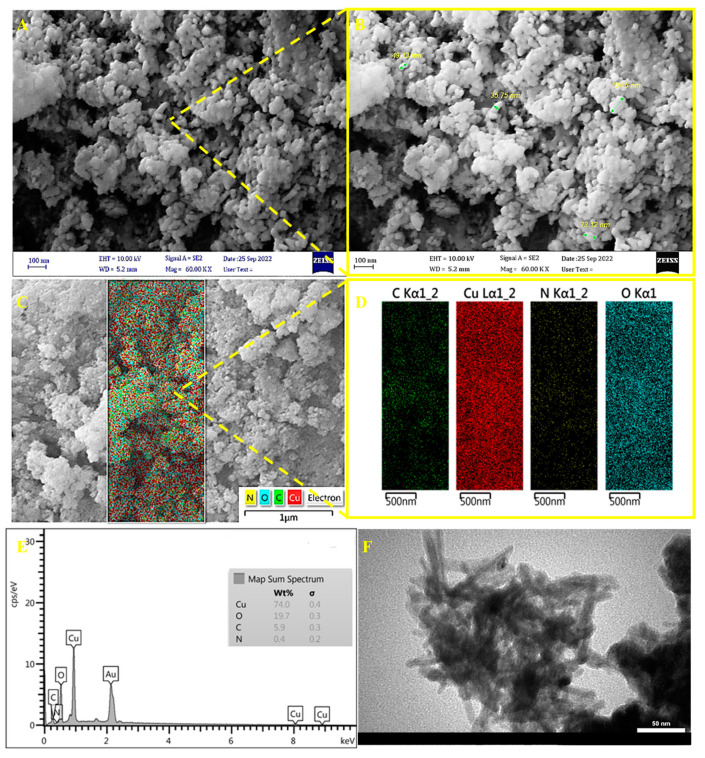
(**A**,**B**) SEM images, (**C**–**E**) EDX analysis and elemental mapping, and (**F**) TEM image of the synthesized Cu-Guo NRs.

The SEM images in Figure 1A,B show that the synthesized Cu-Guo NRs consist of relatively uniform NRs with worm-like shapes with an average size less than 65 nm. EDX analysis and elemental mapping of Cu-Guo NRs in Figure 1C–E highlight the elemental composition, including copper (Cu), oxygen (O), carbon (C), and nitrogen (N) with ratios of 74% (red map), 19.7% (blue map), 5.9% (green map), and 0.4% (yellow map), respectively, according to the raw materials used. The gold (Au) peak is related to the sample preparation process under the SEM instrument. Moreover, the TEM image in Figure 1F confirmed the rod-like shape nanostructures with smooth surfaces and an average diameter of 14 nm. These findings revealed that Cu-Guo NRs was successfully formed with a nano-dimension size according to the literature [34].

**Figure 2:**
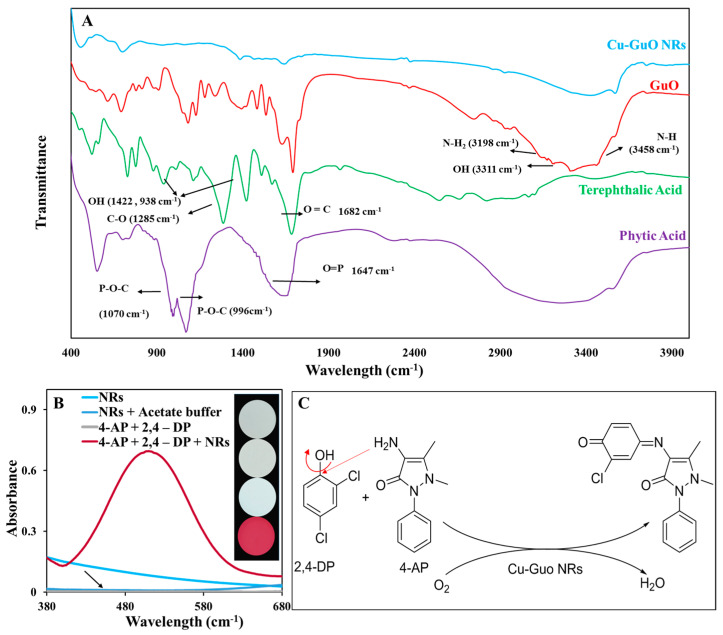
(**A**) FTIR spectra of phytic acid, terephthalic acid, Guo, and Cu-Guo NRs. (**B**) The absorbance spectra of NRs, the mixture of NRs and acetate buffer, and 2,4-DP and 4-AP in the absence and presence of NRs nanozyme with their corresponding images, and (**C**) a proposed reaction mechanism of 2,4-DP and 4-AP catalyzed by Cu-Guo NRs nanozyme.

FTIR spectra of phytic acid, terephthalic acid, GuO, and Cu-Guo NRs were recorded for more investigation. As shown in Figure 2A, the spectrum of phytic acid exhibited a wide bond at 1647 c$m^{-1}$, assigned to the stretching vibration of the P=O bond. A two-branch sharp peak at 1070 c$m^{-1}$ and 996 c$m^{-1}$ was ascribed to the symmetric and asymmetric vibrations of P-O–C bonds in the phytic acid [44]. For terephthalic acid, the sharp bands at 1682 c$m^{-1}$ and 1285 c$m^{-1}$ were related to stretching vibrations of C=O and stretching vibrations of C–O, respectively. Furthermore, bonds at 1422 c$m^{-1}$ and 938 c$m^{-1}$ corresponded to bending vibrations of O–H [45]. The FTIR spectrum of Guo displayed a significant characteristic peak at 3311 c$m^{-1}$, attributed to the stretching vibrations of O–H. The peaks at 3198 c$m^{-1}$ and 3458 c$m^{-1}$ were assigned to NH_2_ and N–H bonds of primary amine, respectively [46]. Comparing all FTIR spectra with Cu-Guo NRs showed that all raw materials, especially Guo, were more crowded with several peaks, while the resulting spectrum dramatically changed by interacting Guo with Cu, and some peaks were removed. The disappearance of some peaks related to CO bonds in Cu-Guo NRs may have been rooted in the fact that the interactions took place through OH groups. The shifting of the vibrations related to phytic acid and terephthalic acid and the change in their signal intensities proved that Cu-Guo NRs were successfully synthesized according to the literature [34]. 

### 3.2. Evaluation of the Laccase-like Catalytic Activity of Cu-Guo NRs

As mentioned, Rtn detection is based on the laccase-like catalytic activity of Cu-Guo NRs. To ensure the beneficial function of this nanozyme, two compounds, including 4-AP (a well-known reagent for phenol estimation) and 2,4-DP (a famous substrate for laccase-based oxidation), were utilized to study the chromogenic reaction between them via Cu-Guo NRs. First, 100 µL of 4-AP (4.9 mM), 100 µL of 2,4-DP (6.1 mM), and 700 µL of acetate buffer (pH = 5.6) were mixed, and then 100 µL of Cu-Guo NRs (1 mg m$L^{-1}$) was added to them and stirred. After 1 h, the obtained mixture was centrifuged, and the corresponding absorbance spectra were recorded. As indicated in Figure 2B, the absorbance spectra of the mixture of 2,4-DP and 4-AP in the absence of Cu-Guo NRs did not present a significant peak in the visible region, while the presence of Cu-Guo NRs exhibited a sharp peak at 508 nm along with a shiny burgundy color. This spectroscopic behavior with a vivid color change from burgundy to colorless may be ascribed to a nucleophilic attack of the NH_2_ group of 4-AP to 2,4-DP under the S_N_2 substitution reaction in the presence of Cu-Guo NRs as the laccase-like enzyme in the mixture according to the proposed possible mechanism in Figure 2C [40]. It can be concluded that Cu-Guo NRs not only have excellent catalytic activity but also work based on the green chemistry principle because of the generation of H_2_O molecule as the only by-product during the reaction.

### 3.3. Principle of the Nanozyme Activity of Cu-Guo NRs in Rtn Detection

**Figure 3:**
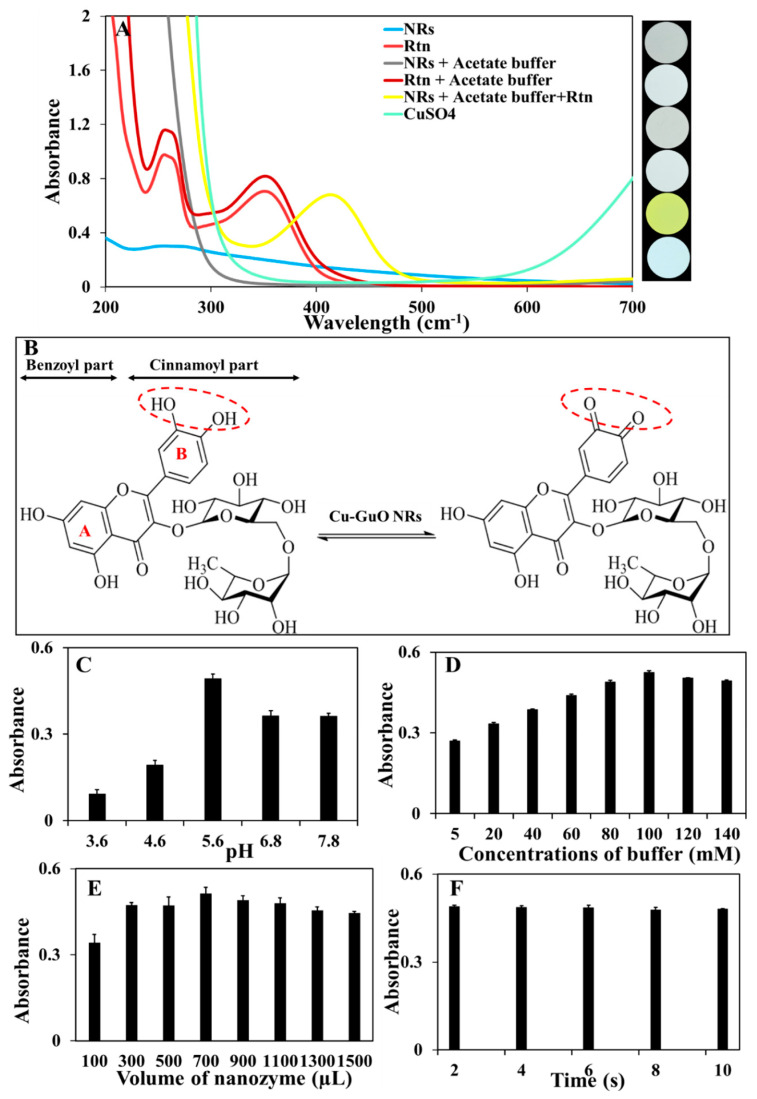
(**A**) UV–Vis spectra of the different solutions: 700 µL of Cu-Guo NRs (1 mg m$L^{-1}$), Rtn (46.68 μM), 700 µL of Cu-Guo NRs (1 mg m$L^{-1}$) in the presence of acetate buffer (100 mM, pH of 5.6); Rtn (46.68 μM) in the presence of acetate buffer (100 mM, pH of 5.6); Rtn (46.68 μM) in the presence of acetate buffer (100 mM, pH of 5.6); and 700 µL of NRs (1 mg m$L^{-1}$) and CuSO_4_ (800 μM) with their corresponding images (from top to down). (**B**) The proposed possible sensing strategy of Rtn based on the catalytic oxidation reaction by Cu-Guo NRs. The effect of the (**C**) buffer type in a solution containing acetate buffer (100 mM) with different pH values of 3.6, 4.6, and 5.6 and HEPES buffer (100 mM) with different pH values from 6.8 to 7.8, in the presence of Rtn (46.68 µM) and 700 µL of Cu-Guo NRs (1 mg m$L^{-1}$). (**D**) Buffer concentration in a solution containing acetate buffer (pH of 5.6) with different concentrations from 5 mM to 140 mM, Rtn (46.68 μM) and 700 µL of Cu-Guo NRs (1 mg m$L^{-1}$). (**E**) Nanozyme volume in a solution containing 100 mM of acetate buffer (pH of 5.6) and various volumes of Cu-Guo NRs from 100 µL to 1500 µL and Rtn (46.68 μM). (**F**) Reaction time from 2 min to 10 min for a solution containing 100 mM of acetate buffer (pH of 5.6) and Rtn (46.68 µM) and 700 µL of Cu-Guo NRs (1 mg m$L^{-1}$).

The main purpose of the study is the introduction of a new strategy for Rtn sensing based on the catalysis reaction of Cu-Guo NRs nanozyme in a relatively acidic condition. Since the hydroxyl groups in the catechol ring of Rtn are susceptible to self-oxidization and convert to ortho-quinone under slightly alkaline conditions [47,48], the catalytic reaction of this nanozyme was accompanied in the acidic medium of acetate buffer. To further investigate the assay principle of Rtn, UV–Vis spectra for each substrate and a mixture of them were recorded at 300 nm to 700 nm. As shown in Figure 3A, Cu-Guo NRs (periwinkle blue color) and a mixture of Cu-Guo NRs with acetate buffer (colorless) did not exhibit a significant absorbance peak. Moreover, CuS$O_{4}$, as a raw material for the synthesis of Cu-Guo NRs, did not illustrate color and sharp bonds at this range. Rtn showed two sharp bonds at 260 nm and 356 nm, as expected for the aromatic rings A and B of flavonoids (Figure 3B) [49,50]. In contrast, adding acetate buffer to Rtn enhanced the signal intensities without any shift at the peak position and did not illustrate color in the 300 nm to 700 nm range. The mixture of Cu-Guo NRs, Rtn, and acetate buffer displayed a sharp peak at 430 nm with a dark yellow color and the same signal intensity compared to Rtn. This red shift (65 nm) of Rtn’s absorbance peak with a vivid color change may be rooted in the fact that the laccase-like catalytic activity of Cu-Guo NRs regarding Rtn in relative acidic condition. Accordingly, it can be expected that two hydroxyl groups in the catechol ring (B) of Rtn are oxidized by Cu-Guo NRs, and the oxidized form of Rtn (o-quinone) results based on the proposed mechanism in Scheme 1B. The developed methodology utilizing Cu-Guo NRs in Rtn catalysis provides a vivid color tonality change from light green to dark yellow, guaranteeing a green and simple visual sensing of Rtn. 

### 3.4. Optimization of the Measurement Conditions

To achieve a stable response, the effect of some effective parameters on the oxidation reaction of Rtn, including the type of buffer, buffer concentration, pH value, nanozyme volume, and reaction time were studied. Since Rtn is slightly oxidized in a basic medium [51], the alkaline condition is unsuitable for the Rtn assay. Therefore, acidic and neutral media were selected for more investigations. Two different types of buffers, including acetate buffer (100 mM) with pH values of 3.6, 4.6, and 5.6 and HEPES buffer (100 mM) with pH values of 6.8 and 7.8 were separately added to a solution containing Rtn (46.68 µM) and 700 µL of Cu-Guo NRs (1 mg m$L^{-1}$), and the corresponding absorbance spectra were recorded after 6 min. The results indicated that 100 mM of acetate buffer with a pH value of 5.6 had the maximum absorbance signal, suggesting this amount of acidity provides the best medium for the nanozyme activity in Rtn oxidation (Figure 3C). To explore the medium ion strength, a series of different concentrations of acetate buffer (pH = 5.6) from 5 mM to 140 mM was tested. The results showed that the absorbance signal was significantly increased by the increasing concentration of a buffer from 5 mM to 100 mM and then gradually decreased (Figure 3D). It was found that an ionic strength of more than 100 mM of the acetate buffer caused a disturbance in the nanozyme activity towards Rtn. Therefore, 100 mM was selected as the optimum concentration of the acetate buffer. Nanozyme volume as an important parameter in Rtn determination was investigated by recording the absorbance spectra of the solution containing 100 mM acetate buffer (pH = 5.6) in the presence of different volumes of Cu-Guo NRs (1 mg m$L^{-1}$) from 100 µL to 1500 µL and 46.68 µM of Rtn. The results revealed that the highest absorbance signal was obtained in 700 µL of Cu-Guo NRs, and less and more than this volume did not prepare a suitable condition for Rtn oxidation (Figure 3E). Thus, 700 µL of Cu-Guo NRs was applied for further experiments. An evaluation of various time intervals over a range of 2–10 min after Rtn addition demonstrated that the corresponding absorbance signal reached a plateau at 6 min, and this time was enough for Rtn catalysis by the nanozyme (Figure 3F). So, 6 min was selected as the optimum reaction time.

### 3.5. Rtn Detection

**Figure 4:**
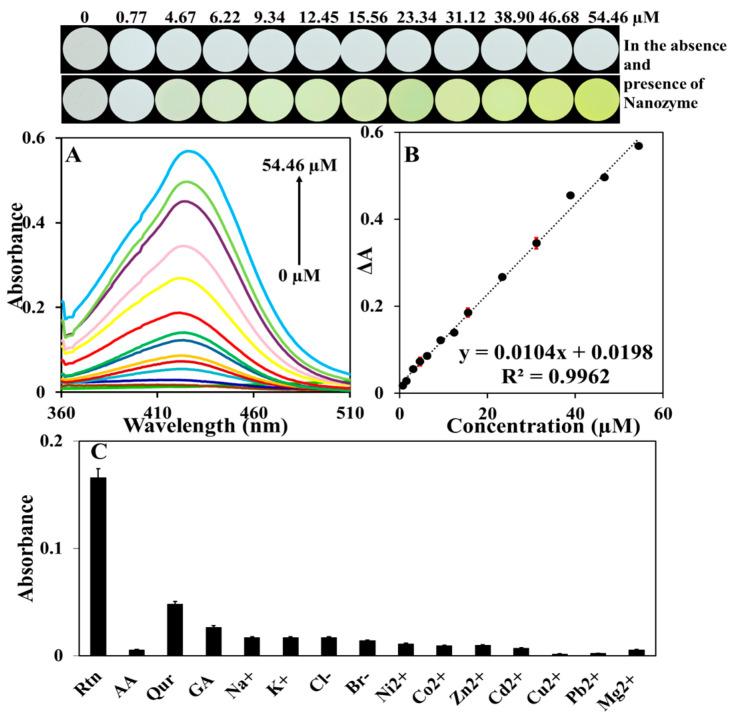
(**A**) UV–Vis spectra of different concentrations of Rtn including 0, 0.77, 1.56, 3.11, 4.67, 6.22, 9.34, 12.45, 15.56, 23.34, 31.12, 38.9, 46.68, and 54.46 µM in the presence of Cu-Guo NRs nanozyme under optimum condition (from bottom to top). (**B**) Calibration curve resulting from the change of the absorbance signal (ΔA) vs. different concentrations of Rtn with low error bar values, along with the recorded images for different concentration of Rtn in the presence and absence of nanozyme. (**C**) The absorbance spectra of the sensor response in the presence of common interfering species such as Qur (33.08 µM), AA (567.79 µM), and GA (235.13 µM) and various ions including $K^{+}$ (2.558 mM), ${\mathrm{Na}}^{+}$ (4.349 mM), ${\mathrm{Cl}}^{-}$ (2.820 mM), ${\mathrm{Br}}^{-}$ (1.251 mM), ${\mathrm{Ni}}^{2+}$ (1.703 mM), ${\mathrm{Co}}^{2+}$ (1.696 mM), ${\mathrm{Zn}}^{2+}$ (1.529 mM), ${\mathrm{Cd}}^{2+}$ (8.895 mM), ${\mathrm{Cu}}^{2+}$ (1.573 mM), ${\mathrm{Pb}}^{2+}$ (0.482 mM), and ${\mathrm{Mg}}^{2+}$ (4.114 mM) in the determination of 16.38 µM Rtn.

To examine the applicability of the proposed sensor regarding Rtn, a series of different concentrations of Rtn was added to the solution, and the absorbance signal difference (ΔA) before and after adding Rtn was recorded at 423 nm under optimum conditions. As shown in Figure 4A,B, the increase of Rtn concentration from 770 nM to 54.46 μM linearly corresponds to the absorption signal increase under a regression equation of ΔA = 0.017 [Rtn] + 0.0198 with a correlation coefficient (R^2^) of 0.9962. The detection limit (LOD) value was calculated to be 114 nM based on S/N = 3, with a relative standard deviation (RSD) value of 1.26% for triplicate. The RSD value (n = 3) of NRs-to-NRs variations (inter-day) for detection of 46.68 µM Rtn was 3.27%, indicating a satisfactory reproducibility method. It is noteworthy that the proposed sensor not only suggested the visual detection of Rtn but also presented excellent linear dynamic range (LDR) and LOD values, which are comparable with other previously reported Rtn sensors, as summarized in Table 1. Another prominent advantage of this study was the rapid and free-enzyme visual measurement of Rtn, which resulted in various color variations under a broader range from light green to light yellow and dark yellow as the naked-eye detection. The results indicated that Cu-Guo NRs had a prominent key role in the visual detection of Rtn, and no vivid concentration-dependent color variations were achieved in the absence of this nanozyme. The herein-described methodology for nanozyme synthesis and its applicability in the visual detection of Rtn is cost-effective, relatively fast, and based on green chemistry principles, which may promise a huge revolution for the Rtn assay in various matrixes.

### 3.6. Selectivity and Anti-Interference Study

To examine the selectivity of the sensor, some common interfering species for Rtn determination were tested. The corresponding spectra for Qur (33.08 µM), AA (567.79 µM), and GA (235.13 µM) and various ions, including $K^{+}$ (2.558 mM), ${\mathrm{Na}}^{+}$ (4.349 mM), ${\mathrm{Cl}}^{-}$ (2.820 mM), ${\mathrm{Br}}^{-}$ (1.251 mM), ${\mathrm{Ni}}^{2+}$ (1.703 mM), ${\mathrm{Co}}^{2+}$ (1.696 mM), ${\mathrm{Zn}}^{2+}$ (1.529 mM), ${\mathrm{Cd}}^{2+}$ (8.895 mM), ${\mathrm{Cu}}^{2+}$ (1.573 mM), ${\mathrm{Pb}}^{2+}$ (0.482 mM), and ${\mathrm{Mg}}^{2+}$ (4.114 mM) were separately recorded under the optimized conditions. The results in Figure 4C reveal that the sensor response for all substances with a concentration more than Rtn (16.38 µM) is negligible, promising acceptable selectivity for the Rtn assay in different complex matrixes.

### 3.7. Real Sample Analysis

The applicability of the developed sensor for Rtn monitoring in some samples such as propolis dry extract, Rtn-containing dietary supplement tablet, human urine, and plasma samples as the real samples were evaluated. The samples were prepared according to the described procedures in the “Preparation of real samples”. First, 200 µL of the obtained samples was separately analyzed by the proposed sensor under the standard addition method, and the Rtn concentration was calculated in each sample. As listed in Table 2, the resulting concentrations agreed with the real concentrations. The concentration of Rtn in the propolis dry extract and dietary supplement tablet were, respectively, 9.42% and 18.38 mg per tablet. The estimated satisfactory recovery confirmed the acceptable feasibility and applicability of the proposed sensor for accurate colorimetric measurements of Rtn.

## 4. Conclusions

A sensitive and enzyme-free colorimetric procedure was proposed for the visual detection of Rtn for the first time. The assay principle mainly relies on the fact that Cu-Guo NRs present a laccase-mimicking nanozyme regarding Rtn and oxidizes Rtn in the relatively acidic conditions by the spectra profile change. The developed methodology facilitated the visual assay of Rtn through the distinct color tonality change from light green to dark yellow and implied the absence of Cu-Guo NRs did not result in a vivid concentration-dependent color variation. The results revealed a broad linearity of the calibration curve for Rtn monitoring with a low LOD compared to other reported sensors and acceptable accuracy in the real samples, including Rtn-containing dietary supplement tablets, propolis dry extract, human urine, and plasma samples. Since the applied strategy by utilizing the laccase-mimicking nanozyme of Cu-Guo NRs for the naked-eye monitoring of Rtn does not need complicated instruments, expensive materials, or skilled personnel, this strategy may promise to extend the portable sensors for the routine analysis of Rtn in complex matrixes.

## Figures and Tables

**Table 1:** Rtn detection based on the proposed colorimetric sensor and other reported sensors.

Material/Probe	Method	LDR (μM)	LOD (μM)	RSD%	pH	Sample	Time	Ref.
Chemically cross-linked Cu-CHIT ^1^/MWCNT ^2^ on GCE ^3^	Electrochemically	0.05–100	0.01	2.43	0.04 M Britton–Robinson buffer (pH = 3)	Orange, lemon, lime, apple	40 s	[1]
Carbon composite electrode modified with Cu (II) phosphate immobilized in a polyester resin	Electrochemically	0.098–2.50	0.011	-	0.1 M phosphate buffer/ethanol (pH = 6.9)	Pharmaceutical formulations	-	[19]
MIP ^4^ with IL ^5^-Gr ^6^	Electrochemically	0.029–1.0	0.001	3.05	0.04 M Britton–Robinson buffer (pH = 2.0)	Tablet	10 min	[25]
Boron-doped diamond electrode	Electrochemically	0.016–0.164	0.0028	3.5	0.1 M Britton–Robinson buffer (pH = 4)	Dietary supplement products	1 min	[21]
Polyphenol oxidase/AuNPs ^7^/mesoporous carbon-modified electrode	Electrochemically	1.50–28.00	0.51	4.6	0.1 M phosphate buffered saline (pH = 2)	Dark teas	-	[22]
Cu-zeolite A/Gr on GCE	Electrochemically	0.23–2500	0.12	1.1–2.1	oxalate buffer (pH 4.5)	Urine	-	[27]
GO-CHIT/GCE	Electrochemically	0.9–90	0.56	1.81	citrate buffer (pH = 4.5)	Fruits	25 s	[52]
MIP/MWCNTs/GCE	Electrochemically	0.4–10	0.11	3.01	buffer (pH = 8.0)	Tablet	10 min	[53]
Mobile phase	HPLC	1.64–819.0	0.16	-	-	*Ginkgo biloba* L. Leaves	40 min	[23]
Mobile phase	HPLC	0.82–40.95	0.16	-	50 mM buffer KH_2_PO_4_ (pH = 4.6)	Blood plasma, cardiomyocytes, and mitochondria of cardiomyocytes	6–6.5 min	[26]
CDs ^8^	Spectrofluorimetry	0.30–9.16	0.098	-	-	Tablet	-	[24]
Rutin-Al (III) complex	Spectrophotometry and fluorimetry	4.09–36.85 and 6.55–39.31	0.11 and 0.12	1.06 and 1.40	acetic acid buffer (pH = 4.8)	Tablet	-	[20]
Complex with ytterium (III)	Fluorimetry	0.02–1.96	0.0021	1.6	Tris-HCl buffer (pH = 9.5)	Tablet	-	[8]
Cu-Guo NRs	Spectrophotometry and visually	0.77–54.46	0.114	1.26	acetate buffer (pH = 5.6)	Blood plasma, urine, tablet, dry propolis extract	6 min	This Study

^1^ Chitosan, ^2^ multi-walled carbon nanotubes, ^3^ glassy carbon electrode, ^4^ molecularly imprinted poly, ^5^ ionic liquid, ^6^ graphene, ^7^ gold nanoparticles, ^8^ carbon dots.

**Table 2:** Determination of Rtn in various real samples by the developed sensor.

Sample	Regression Equation	R^2^	Added/ Contained (µM)	Found (µM)	Recovery %	RSD
Tablet	A = 0.0166 X + 0.0383	0.9989	0 8.19 16.38	3.78 12.33 19.82	- 104.4 97.9	2.41 1.89 2.09
Propolis	A = 0.0156 X + 0.0981	0.9991	0 8.19 16.38	10.29 18.39 25.96	- 99 95.7	2.03 3.26 4.48
Urine (1)	A = 0.0211 X + 0.0014	0.9992	0 8.19 16.38	N.D. 8.43 15.95	- 103 97.4	- 3.21 2.49
Urine (2)	A = 0.0199 X + 0.002	0.999	0 8.19 16.38	N.D. 8.45 15.95	- 103.2 97.4	- 2.55 3.93
Plasma	A = 0.0180 X + 0.0042	0.9996	0 8.19 16.38	N.D. 8.075 16.72	- 98.66 102.11	- 1.92 3.47

## Data Availability

Not applicable.
